# Bryozoan zooid size variation across a bathymetric gradient: a case study from the Icelandic shelf and continental slope

**DOI:** 10.1007/s00227-017-3231-9

**Published:** 2017-09-07

**Authors:** Anna Stępień, Piotr Kukliński, Maria Włodarska-Kowalczuk, Małgorzata Krzemińska, Gudmundur Gudmundsson

**Affiliations:** 1 Institute of Oceanology Polish Academy of Science, Powstańców Warszawy 55, 81-712 Sopot, Poland; 2 The Icelandic Institute of Natural History, Urriðaholtsstræti 6-8, 210 Garðabær, Iceland

## Abstract

Body size is one of the most important biological characters, as it defines many aspects of organismal functioning at the individual and community level. As body size controls many ecological aspects of species, it is often used as a proxy for the status of the ecosystem. So far no consistent mechanism driving size shift has been proposed. In this study, we investigated bathymetric variability in zooid’s size and shape in aquatic colonial animals, Bryozoa. Although the response of bryozoan zooid size to temperature or food concentration has been experimentally proven, the effects of natural environmental variability on marine bryozoan populations has been much less explored. The presented investigation is aimed to assess the bathymetric patterns and environmental drivers of bryozoan zooid size on continental shelf and slope of southern Iceland. 196 colonies of 11 species representing different colonial forms and taxonomic groups were selected for zooid characteristics measurements. A pattern of depth-related increase in zooid size was documented for *Bicellarina alderi*, *Chartella barleei* and *Sarsiflustra abyssicola*, no statistically significant effects were detected for the other eight species. Two species *Bicellarina alderi* and *Caberea ellisii* had significantly longer zooids in deeper water, shape of the remaining species did not change along the bathymetric gradient. Intercolonial coefficient of variation in zooid size did not change across the depth gradient. Temperature differences along studied depth could be responsible for the observed pattern.

**Electronic supplementary material:**

The online version of this article (doi:10.1007/s00227-017-3231-9) contains supplementary material, which is available to authorized users.

## Introduction

Body size is considered as one of the most important adaptations to the external environment (Hunt and Roy 2006). It has been shown that size influences both the biological (e.g., metabolism rate, growth rate) and the ecological (e.g., community organization) aspect of individual, population and multispecies community functioning (McClain and Rex 2001; Smith and Brown 2002). In fact, size changes in one group can have dramatic consequences on the functioning of entire food webs (Yvon-Durocher et al. 2011). Therefore, phenotypical responses to environmental conditions have been recognized to be of prime importance to investigate (e.g., Rex and Etter 1998; Roy 2002; Smith and Brown 2002; Atkinson et al. 2006).

Bathymetric transects across continental margins, from shelf to abyssal plains, are characterized by steep gradients. Here, several factors, including water temperature, salinity, oxygen concentration, food availability, and sediment stability can change dramatically with depth (Thistle 2003). Therefore, bathymetric gradients can serve as suitable ‘natural laboratory’ settings for testing various ecological hypotheses, including those related to an organism’s body size. It has already been suggested that body size may be influenced by a combination of factors, and that the driving mechanisms, and any resulting patterns, may vary across taxonomic and functional groups (Smith and Brown 2002; Collins et al. 2005). Recognizing the environmental factors which are responsible for body size adaptations associated with bathymetric gradients, could provide not only an understanding of natural body size variability, but could also facilitate the prediction of environmental changes that are likely to occur as a result of on-going climate warming. In fact, the reduction in body size displayed by some organisms is considered as one of the most important biological responses to global warming (Sheridan and Bickford 2011). Body size reduction may affect biological productivity and energy flow, as well as alter the functionality of food webs (Gardner et al. 2011). Therefore, understanding the patterns and mechanisms of adaptive body size variations is a priority for present-day ecological investigations.

Trends in body size changes across bathymetric gradients have been investigated in conjunction with the discovery of high species richness in the deep-sea (Rex and Etter 1998; Roy 2002; Smith and Brown 2002; Olabarria and Thurston 2003; McClain 2004; Udalov et al. 2005). Both gigantism (most pronounced in crustaceans) and dwarfism were documented for a variety of taxonomic groups (Timofeev 2001). Several explanation were proposed for the observed trends (Thiel 1975; Sebens 1982; Rex and Etter 1998). For example, Thiel (1975) suggested that size among deep-sea organisms is shaped by three main factors: food availability, metabolic rates (which are driven by temperature), and constraints in reproductive success. He suggested that evolution in the deep-sea may have favored a smaller body size because of limited food supplies and a tendency to a higher reproductive success in dense populations of smaller organisms, as compared to populations consisting of larger but more sparsely distributed individuals.

Another explanation—known as the ‘Optimal Size Theory’—was proposed by Sebens (1982) who suggested that optimal body size occurs when the differences between energetic costs and food intake are greatest. Therefore, body size should decrease with depth as prey distribution becomes progressively patchier (i.e., prey availability becomes lower), thereby increasing the energetic costs of foraging. Rex and Etter (1998) tested this model while investigating depth-related changes in body size for several gastropod species. Contrary to Sebens’ hypothesis, they found that the deepest occurring gastropod increased in size with depth, and concluded that, in this case, a larger body size may be competitively advantageous.

The present investigation focuses on bryozoans, aquatic colonial organisms composed of asexually budded modular units termed zooids (Ryland 2005). The size of a bryozoan zooid is believed to have ecological and physiological significance. For example, it was suggested that having very large zooids is advantageous in interspecific competition for space (Grischenko et al. 2002). In addition, the size of the lophophore, an organ responsible for capturing food particles, increases proportionally with zooid size (McKinney and Jackson 1991). Therefore, species with larger zooids appear to be better competitors because of their ability to produce more powerful feeding currents (Grischenko et al. 2002).

**Fig. 1:**
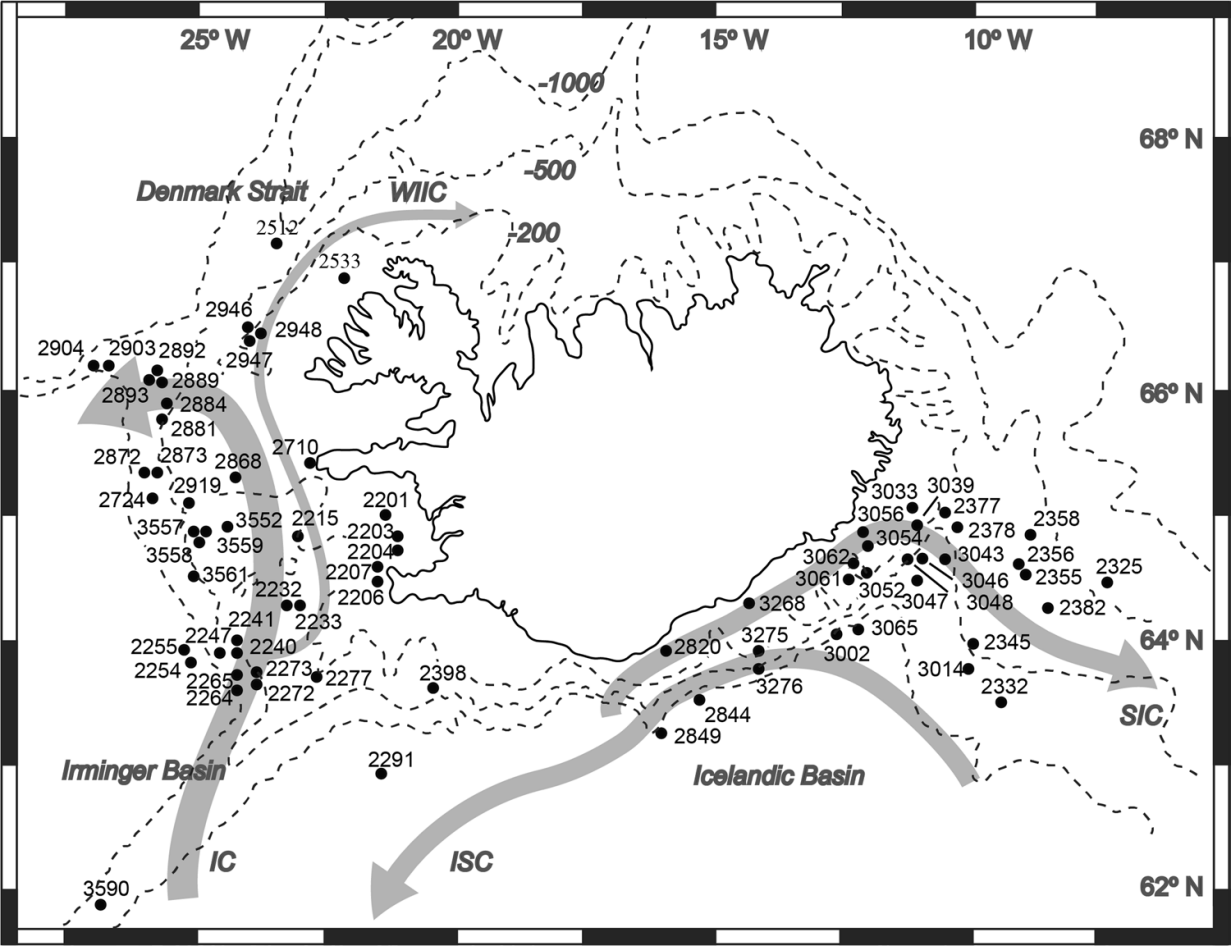
Study area and location of sampling station. *Gray arrows* indicate currents flowing along South Icelandic shelf and slope (*SIC* South Icelandic Current, *ISC* Icelandic Slope Current, *IC* Irminger Current, *WIIC* West Icelandic Irminger Current). *Dashed lines* indicate isobaths (200, 500 and 1000 m)

We explored the patterns of zooid size variability in one bryozoan order—the Cheilostomata, which inhabits the Icelandic shelf and continental margin. In particular, we investigated depth-related environmental factors as potential drivers of zooid body size changes across a bathymetric gradient. The study area’s ecosystem is shaped by an interplay of seabed morphology and strong oceanic currents transporting water masses to and from the Icelandic and Irminger Basins. These two basins are separated by shallow ridges (see Fig. 1). Here, the directional flow of local currents causes nutrients to accumulate in deeper waters (Meißner et al. 2014). Hence, Icelandic deep-sea habitats are relatively rich in organic matter, in contrast to benthic habitats located in the open ocean (Meißner et al. 2014). As food supply in our study area does not significantly decline with depth (Meißner et al. 2014 Figs. 3, 4, 5; Table 2; Ostmann et al. 2014, Table 1), we assume water temperature to be the main factor influencing bryozoan body size across the Iceland continental margin.

**Table 1:** Water temperature, salinity together with total organic carbon at the investigated depth interval

Depth (m)	Temperature (°C)	Salinity	Total organic carbon content (%)	
	South Icelandic water		Irminger Basin	Icelandic Basin
Surface	10–11	35.1–34.7		
100	8–9	35.0–34.7		
200	7–8	35.0–34.8	7.23	
300–500	7	35.0–34.8	4.51	
600–800	6	34.9	4.85	
>1000	5	34.9	3.34–4.74	4.86–9.25

Several characteristics of cheilostomes make them particularly suitable for this kind of investigation: (1) the zooid’s skeletal walls do not change in length or width after budding, at the initial stage of colony development (O’Dea and Okamura 2000a); therefore, in contrast to many other solitary taxa, it is easy to determine whether or not the organism being observed is fully developed, (2) many zooids (often thousands) occur in one colony, thus providing a number of measurable replicates (Ryland 2005) for a more robust and reliable statistical analysis, (3) bryozoan species are common and abundant in a wide range of marine habitats (O’Dea and Okamura 2000a) and at a variety of depths (Clarke and Lidgard 2000) making sampling across environmental gradients relatively easy.

Changes in bryozoan zooid body size have been documented to occur in space and time, and have been interpreted as adaptations to different temperature regimes (O’Dea and Okamura 2000a, b). Most studies reported an inverse body size-temperature relationship. Therefore, zooid body size was proposed as a tool for investigating seasonality in ancient environments (O’Dea and Jackson 2002; O’Dea 2003). Furthermore, laboratory studies supported the hypothesis that temperature influences zooid body size. Amui-Vedel et al. (2007) observed significantly longer and wider *Cryptosula pallasiana* zooids at 14 than at 18 °C. Similarly, Hunter and Hughes (1993, 1994) found that *Cellopora hyalina* had longer zooids at lower temperatures. Menon (1972) obtained similar results after testing differences in zooid size in three species (*Membranipora membranacea*, *Electra pilosa* and *Conopeum reticulum*) kept at four temperatures (6, 12, 18 and 22 °C). Yet, to date, there is no study which investigates variability in bryozoan zooid body size across a natural depth-related thermal gradient.

In this study, we test whether the temperature drop accompanying a depth gradient, together with other depth-associated environmental factors (e.g., light and pressure) will lead to a change in zooid body size in cheilostome bryozoans. We also test whether decreasing seasonal variation with water depth will have an influence on zooid body size. O’Dea and Okamura (1999) showed that seasonal temperature differences are related to variability in zooid body size. In our study area, seasonal variations in environmental factors, especially water temperature, are greater in shallower areas (Holliday et al. 2006; Logemann et al. 2013). We assume this should be reflected in zooid size characteristics—more variable in shallow waters than in the deep-sea, where little seasonal variability occurs.

We hypothesize that: (1) zooid body size and surface area will increase with depth in response to decreasing water temperatures, (2) the variability in zooid body size within same-depth populations will decreases across the bathymetric gradient, from shelf to abyssal habitats, in response to decreasing levels of seasonal variability. We also aim to document depth-related size and shape patterns, which will provide a good baseline for future laboratory experiments or more targeted investigations testing the influence of particular environmental variables.

## Study area

The study area includes the Icelandic and Irminger Basins (Fig. 1). These basins are located off southern Iceland, and are under the influence of warm Atlantic water masses transported from lower latitudes by the North Atlantic Current (Astthorsson et al. 2007). In the Icelandic Basin, the South Icelandic Current (SIC) transports warm water masses in an easterly direction along the shelf. At 500–1100 m depths, the Icelandic Slope Current (ISC) flows in a westerly direction (Meißner et al. 2014). In the Irminger Basin, the strong Irminger Current (IC) flows along the continental slope, while the West Icelandic Irminger Current (WIIC) carries water masses northward through the Denmark Strait. In the southern region, mean surface water temperatures, averaged over 10 years (1990–2000), were 10–11 °C (Malmberg and Valdimarsson 2003) and decreased with depth, being 8–9 °C at 100 m, 7–8 °C at 200 m, 7 °C at 300–500 m, 6 °C at 600–800 m, and 5 °C at 900–1000 m (Hansen and Østerhus 2000; Malberg and Valdimarsson 2003). Salinity varied slightly with depth (34.7–35.2, Holliday et al. 2006). In our study area, Holliday et al. 2006 reported seasonal differences in temperature and salinity on the shelf, based on investigations carried out in 2001–2002: at a depth of 100 m, temperatures/salinity changed from 10 °C/34.7 in spring/summer to 7 °C/35.2 in autumn/winter. Seasonal differences in temperature diminished gradually with depth, and, at 500 m, temperature remained the same (7 °C) regardless of season. At 1000 m, salinity ranged between 34.8 in spring/summer and 35.0 in autumn/winter (Holliday et al. 2006; Logemann et al. 2013).

The Icelandic continental margin is recognized as a highly productive system (ICES 2008; Meißner et al. 2014; Ostmann et al. 2014). Although it is assumed that most of the produced organic matter is consumed in the upper 200 m of the water column, organic matter content in Icelandic deep-sea basin sediments is relatively high. Organic matter is pushed off the ridges by strong currents to the deeper part of the basin (Meißner et al. 2014). Furthermore, bottom currents mediate the dispersion of nutrients along the slope. Meißner et al. 2014, reported that, in the Irminger Basin, continental shelf Total Organic Carbon (TOC) content in sediments was 7.2% (based on data from two stations at 200 m depth), while shelf break TOC was 4.9% (one station at approx. 300 m depth), and slope TOC was 4.5% (two stations at 700 m depth). In the Icelandic Basin deep-sea habitats (more than 900 m), TOC content in sediments ranged from 4.9 to 6.7% (data from five stations) (see Table 1).

## Materials and methods

### Samples

This study is based on selected samples from the BioIce Project collection, which is stored at the Icelandic Institute of Natural History. Samples were collected during a ten-year investigation (1992–2002) that aimed at a comprehensive assessment of benthic invertebrates’ distribution and diversity in the Icelandic Marine Economic Zone (Guðmundsson 2000). BioIce sampling was conducted during 17 cruises aboard two research vessels (*R/V Bjarni Sæmundsson* and *R/V Håkon Mosby*). Samples were collected at several sampling stations located on both shelf and continental margin all around Iceland, covering a wide range of depths (from 20 to 3000 m).

We analyzed a subset of 78 samples including colonies from 11 bryozoan species, collected at depths ranging from 30 to 1000 m (Table 2). Only samples in which at least four colonies per species were found were selected for analysis (Online Appendix 1). The 11 species used for this study represented seven families occurring at a relatively wide depth-range, and representing two different colony forms; four species with encrusting colonies: *Porella struma* (Norman, 1868) (Brycryptellidae), *Ramphonotus minax* (Busk, 1860) (Calloporidae), *Escharina boreale* Hayward, 1994 (Escharinidae), *Escharella abyssicola* (Norman, 1869) (Romancheinidae) and seven species with flexible colonies: *Bicellarina alderi* (Busk, 1959), *Dendrobeania decorata* (Verrill, 1879), *Dendrobeania fruticosa* (Packard, 1863) (Bugulidae), *Caberea ellisii* (Fleming, 1816), *Tricellaria ternata* (Ellis and Solander, 1786) (Candidae), *Chartella barleei* (Busk, 1860), *Sarsiflustra abyssicola* (Sars G. O., 1872) (Flustridae).

**Table 2:** Characteristics of material used for the study

	Depth range (m)	Temperature range (°C)	Total number of colonies	Total number of measured zooids	Colony form
*Bicellarina alderi*	100–1066	7.39–4.21	16	320	F, B
*Caberea ellisii*	100–554	7.31–6.28	20	400	F, B
*Chartella barleei*	100–1111	7.64–4.18	20	400	F, M
*Dendrobeania decorata*	100–554	7.36–5.59	24	480	F, B
*Dendrobeania fruticosa*	121–228	7.69–6.41	13	260	F, B
*Escharella abyssicola*	114–356	7.66–5.54	21	10	E
*Escharina boreale*	140–549	7.76–6.07	14	280	E
*Porella struma*	219–550	6.44–6.34	16	320	E
*Ramphonotus minax*	130–998	7.84–3.78	25	500	E
*Sarsiflustra abyssicola*	300–1085	7.13–4.44	16	320	F, M
*Tricellaria ternata*	227–554	6.44–6.29	14	280	F, B

Depth and temperature range according to BioIce project data

Colony form: *F* flexible, *B* branched, *M* multiserial, *E* encrusting (at least four colonies were used at the depth intervals)

### Measurements

In the case of encrusting species, only colonies which developed on flat surfaces were studied. Colonies were photographed under the stereomicroscope with the use of a digital camera (Nikon D3100). From the pictures, 20 autozooids (modules responsible for feeding) per colony were randomly chosen for measurements. Random selection of zooids for measurements was performed with the use of CPCe software (Kohler and Gill 2006), which distributed points within a specified border; in this case—within colony, but outside of the initial colony development zone, where zooids are smaller than zooids budded during later stages of astogeny (Yagunova and Ostrovsky 2008). Deformed zooids, as well as zooids at bifurcations, were avoided. Zooid morphometric characteristics, namely length (measured along an axis of symmetry), and width (measured along the widest axis perpendicular to the axis of symmetry) were measured using ImageJ software (Rasband 2007). Such software calculates the number of pixels found along a defined vector (Rasband 2007). A millimeter-scaled graticule microscope slide was used for calibration. To determine size characteristics, length and width were used to calculate zooid surface area (length × width). We did not use width alone, as previous papers suggested it to be an unreliable measurement (O’Dea and Okamura 1999). In fact, width is less sensitive to environmental variability than length and area, and it can be strongly influenced by the position of the zooid in the colony (O’Dea and Okamura 1999; Lombardi et al. 2006). For shape characteristics, a Zooid-Shape index (Z-SI, length/width) was used, with larger values indicating a more elongated zooid.

### Data analyses

For each colony of each species, the mean and standard deviation of length, area, and Z-SI were calculated. Relationships between water depth and mean zooid length, surface area and Z-SI (average values for each colony) were explored using Spearman correlation analysis. To examine the patterns of variability in zooid body size among colonies, the coefficient of variation (CV; defined as the standard deviation divided the mean) of zooid length and surface area was also calculated for each colony. The relationships between water depth and CVs for zooid length and surface area, and average values of Z-SI for each colony were explored using Spearman correlation analysis.

## Results

**Fig. 2:**
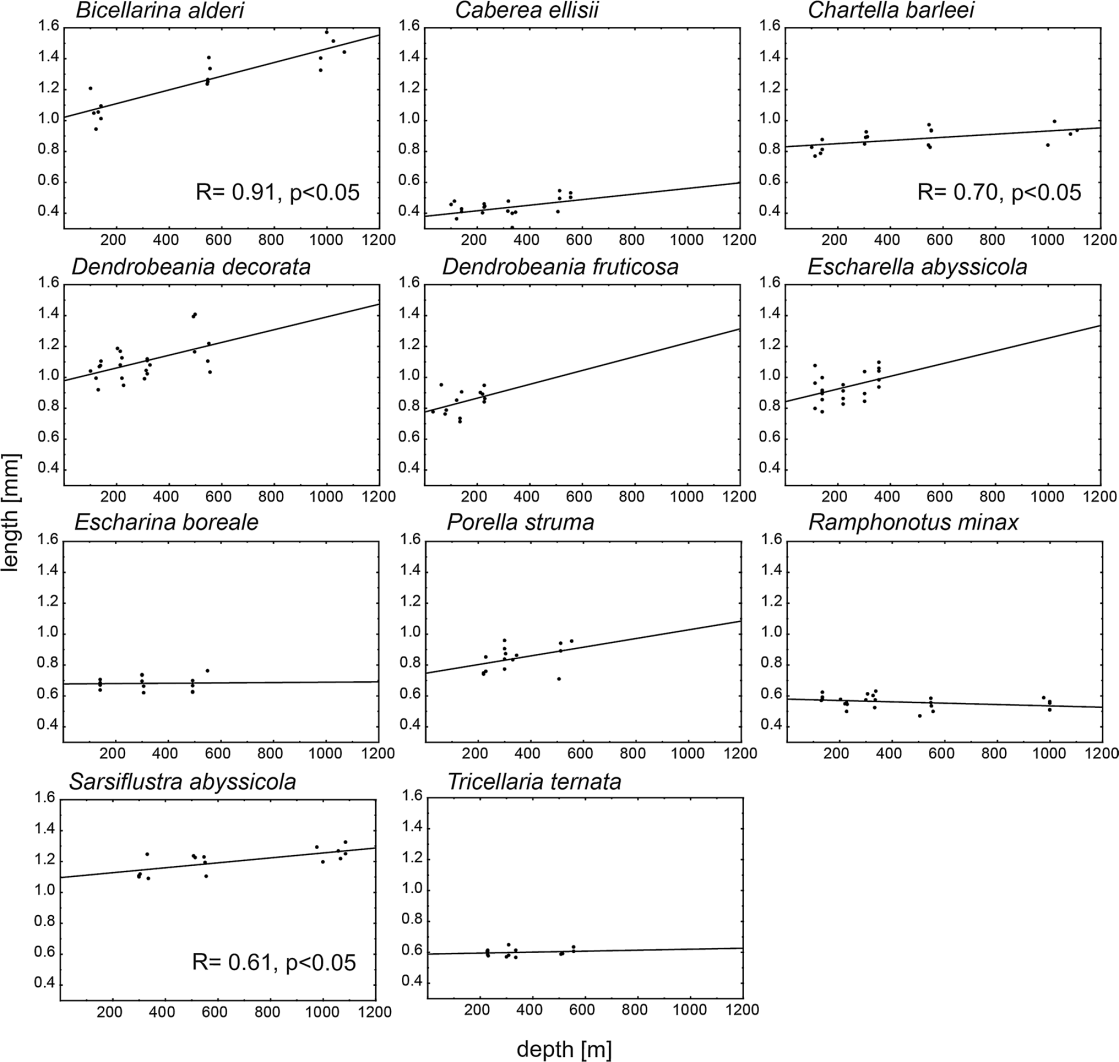
Correlation between length and depth for all species. Each *point* represents mean length of zooids calculated for each colony

**Fig. 3:**
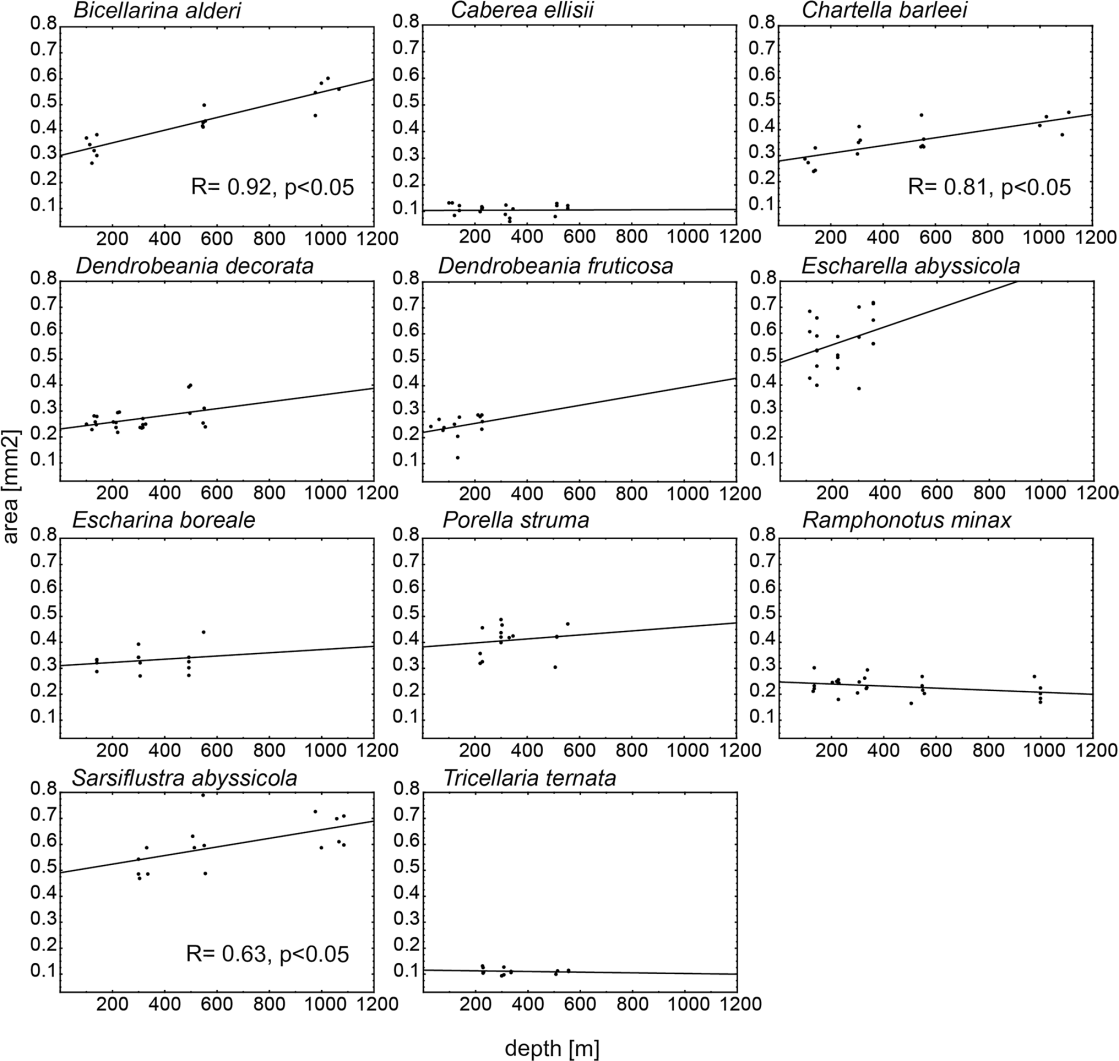
Correlation between area and depth for all species. Each *point* represents mean area of zooids calculated for each colony

There was no consistent water-depth-related pattern in zooid body size for all species studied. There was a significant positive correlation (Spearman correlation analysis, *P* < 0.05) between zooid size and depth for three species: *Bicellarina alderi*, *Chartella barleei* and *Sarsiflustra abyssicola* (Table 3; Figs. 2, 3). Both zooid length and surface area in these species increased with water depth. These three species were characterized by a flexible growth-form and were represented by materials collected from a wide range of depths (100–1000 m; Table 2). The highest correlation coefficient (R) for both length and surface area was documented for *B. alderi* (*R* = 0.90 and 0.92, respectively, *P* < 0.01, Table 3).

**Table 3:** Result of Spearman correlation analysis between zooid characteristics and depth

	Spearman corelation R				CV area	Colony form
	Length	Area	Shape	CV length		
*Bicellarina alderi* ^b^	*0.91*	*0.92*	*0.51*	−0.28	−0.18	F
*Caberea ellisii* ^a^	0.38	−0.04	*0.69*	0.11	0.04	F
*Chartella barleei* ^b^	*0.70*	*0.81*	−0.46	0.02	0.13	F
*Dendrobeania decorata* ^a^	0.40	0.24	0.37	−0.21	−0.29	F
*Dendrobeania fruticosa* ^a^	0.32	0.40	0.32	0.31	−0.15	F
*Escharella abyssicola* ^a^	0.36	0.26	0.41	0.12	−0.15	E
*Escharina boreale* ^a^	−0.09	0.04	−0.24	0.41	0.5	E
*Porella struma* ^a^	0.50	0.31	0.40	0.24	−0.25	E
*Ramphonotus minax* ^b^	−0.36	−0.29	−0.05	0.27	0.06	E
*Sarsiflustra abyssicola* ^b^	*0.61*	*0.63*	−0.08	−0.37	−0.18	F
*Tricellaria ternata* ^a^	0.18	−0.04	0.31	0.15	0.27	F

Significant changes marked by italics (*P* < 0.05)

^a^ Colony found at depth range 50–500 m

^b^ Colonies found at depth range 100–1000 m

There was no significant correlation (Spearman correlation analysis, *P* > 0.05) between zooid body size and depth for the other species: *Dendrobeania decorata*, *Dendrobeania fruticosa*, *Porella struma*, *Caberea ellisii*, *Escharina boreale*, *Escharella abyssicola, Tricellaria ternata* and *Ramphonotus minax* (Table 3). However, for the majority of them (*D.decorata*, *D. fruticosa*, *P. struma*, *C.ellisii*, *E. abyssicola* and *T. ternata*), there was a tendency toward an increase in zooid length with increasing water depth (Fig. 2). In two species (*E. boreale* and *R. minax*) zooid length slightly decreased with increasing depth. Trends toward an increasing zooid surface area with water depth (not supported by a significant correlation) were observed in five species (*D. decorata*, *D. fruticosa*, *P. struma*, *E. boreale*, and *E. abyssicola*), while a slight decline in surface area was noted for the three remaining species (*C. ellisii*, *T. ternata* and *R. minax*; Fig. 3). The species for which no significant correlation was documented were sampled based on materials collected at depths ranging 50–500 m.

**Fig. 4:**
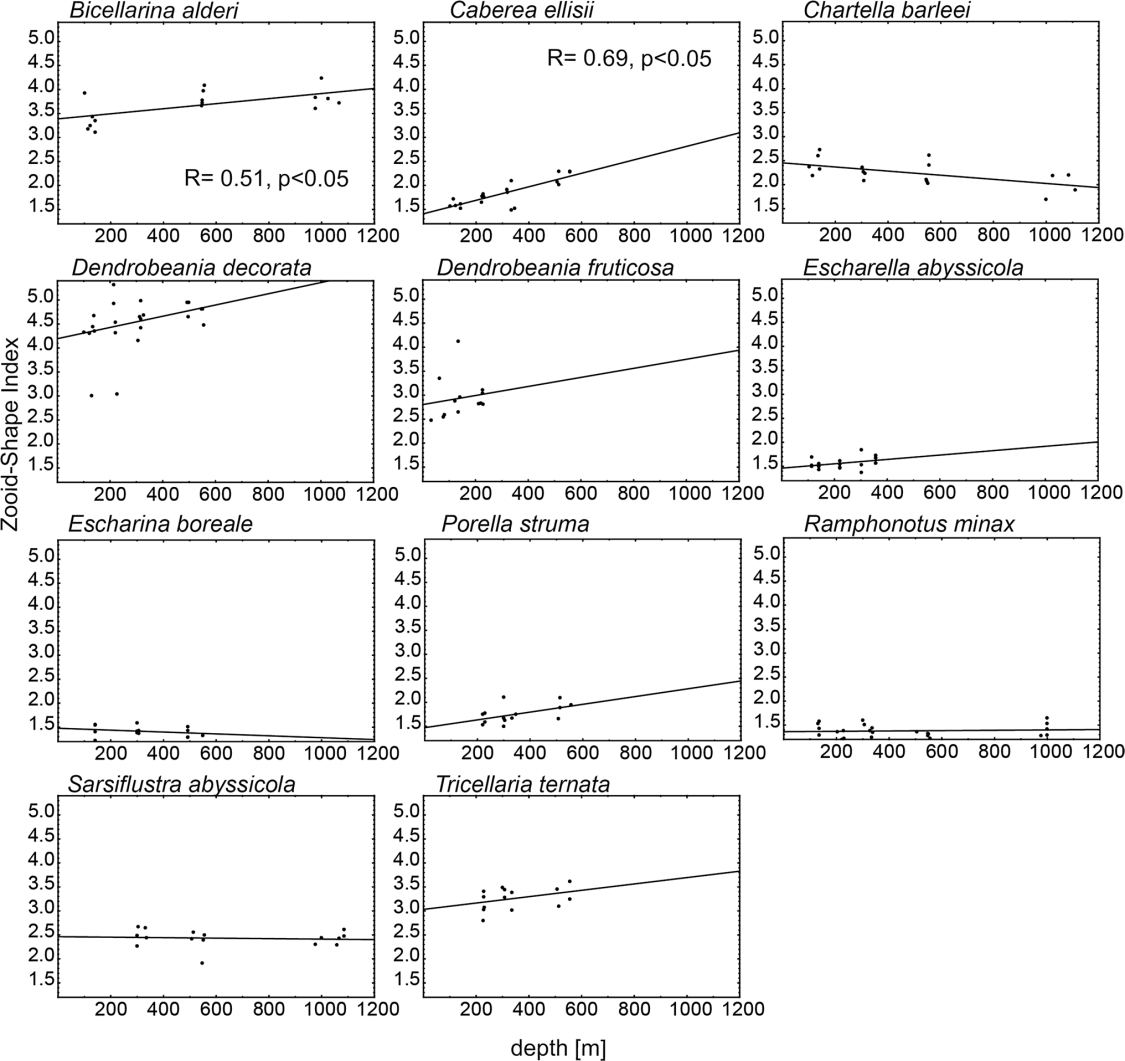
Correlation between Zooid Shape index and depth for all species. Each *point* represents mean index of shape of zooids calculated for each colony

Zooid body shape was significantly correlated with water depth in two species with flexible and branching colonies: *B. alderi* and *C. ellisii* (Table 3; Fig. 4). Zooids in these species were more elongated the deeper the water. There was a tendency toward shorter zooids in deeper water (not supported by a significant correlation) for *C. barleei*, *S. abyssicola*, *E. boreale* and *R. minax* (Fig. 4). Two species: *C. barleei* and *S. abyssicola* had flexible, colonies, while *E. boreale* and *R. minax* had encrusting colonies (Table 2). Trends toward more elongated zooids with water depth were noted for three species with flexible and branching colonies (*D. decorata*, *D. fruticosa*, and *T. ternata*) and for two species with encrusting colonies (*E. abysicola* and *P. struma*; Table 2).

**Fig. 5:**
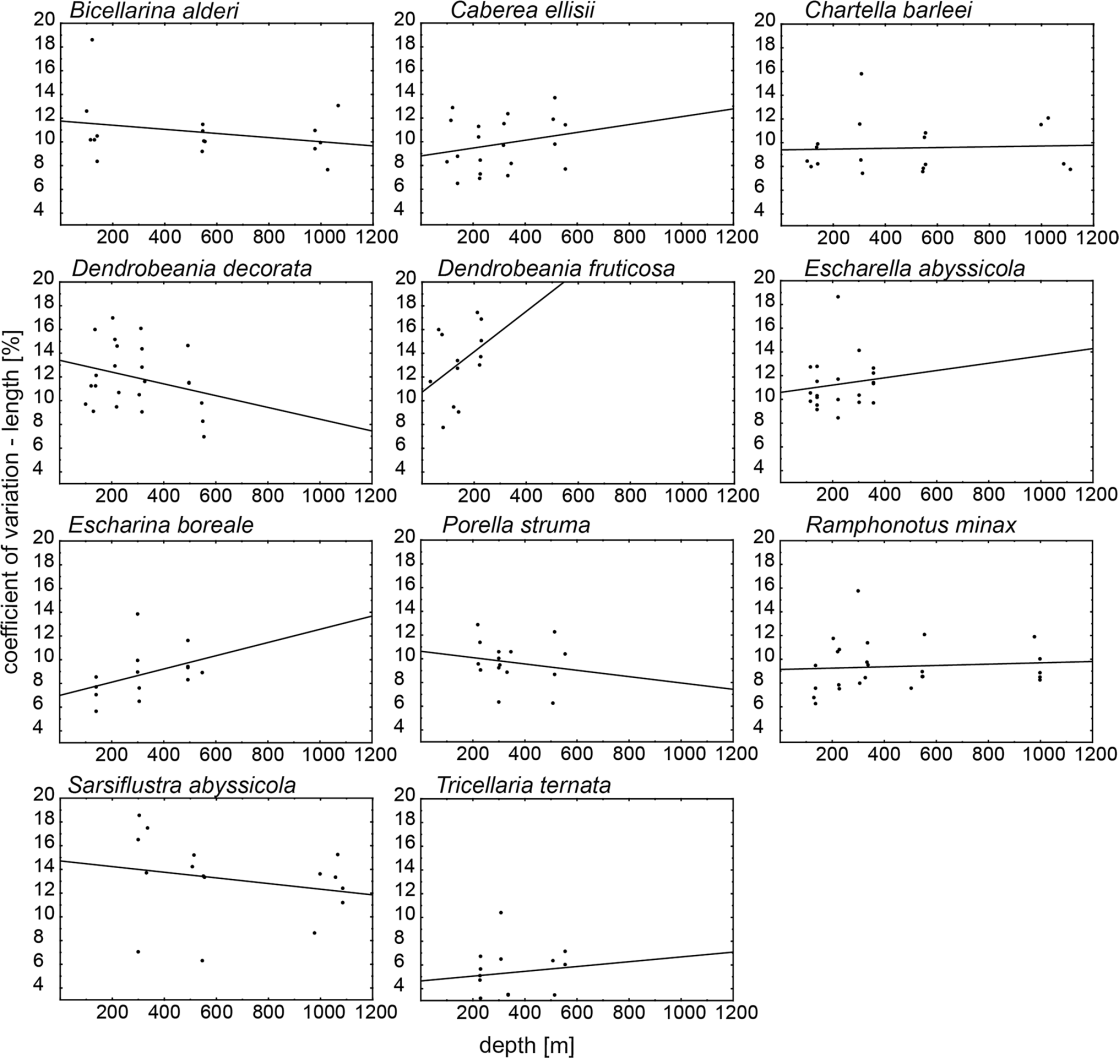
Correlation coefficient of variation (CV) of length and depth for all species. Each *point* represents mean CV of length of zooids calculated for each colony

**Fig. 6:**
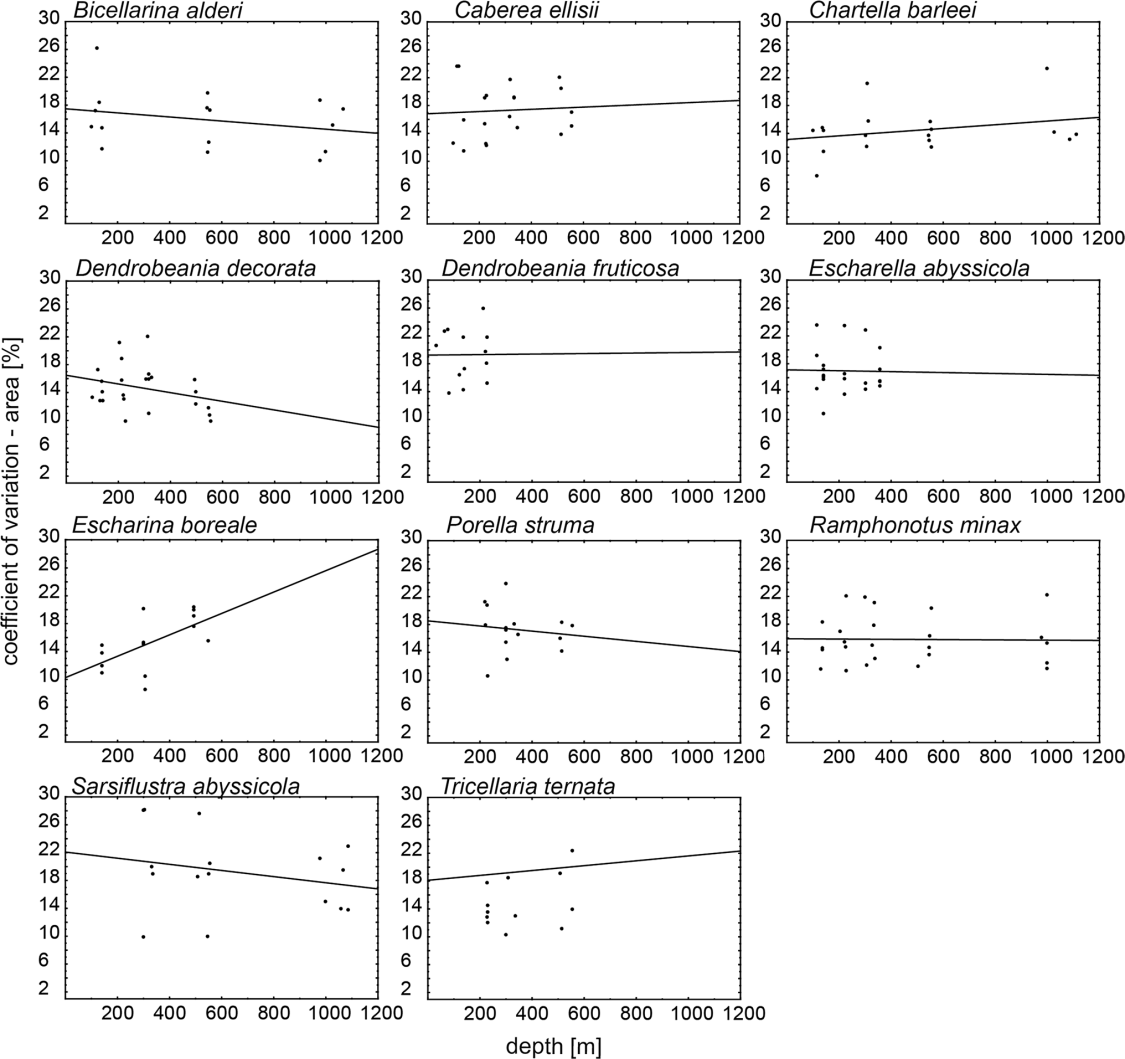
Correlation coefficient of variation (CV) of area and depth for all species. Each *point* represents mean CV of area of zooids calculated for each colony

No significant correlation (Spearman correlation analysis, *P* > 0.05) between colonial coefficient of variation (CV) in length or in area versus depth was documented (Table 3; Figs. 5, 6). Although results did not support a significant correlation, CVs for zooid length showed a trend toward shorter zooids in deeper water for three species (*B. alderi*, *S. abyssicola*, *D. decorata*, and) and a trend toward longer zooids in deeper water for the eight remaining species (Figs. 5, 6). Similarly, CVs for zooid surface area showed a trend toward a smaller surface area with increasing water depth in six species (*B. alderi*, *S. abyssicola*, *D. decorata*, *E. abyssicola* and *D. fruticosa*) and the reverse trend for the five remaining species.

## Discussion

This study did not reveal any consistent patterns of change in bryozoan zooid length, surface area or shape across the investigated depth range. We recorded a statistically significant increase (by about 20%) in zooid size characteristics (length, area) with increased water depth in three species (e.g. *B. alderi*, *C. barleei*, and *S. abyssicola*), while, in the remaining eight species, no statistically significant trend was observed (increase was less than 15%; see Table 4). The species that showed a positive correlation were sampled at a wide range of depths (100–1000 m), where temperatures decreased by 5–6 °C (Malmberg and Valdimarsson 2003; Hansen and Østerhus 2000), while other factors, including salinity and food supply, only decreased slightly (Ostmann et al. 2014). The species for which the correlation was not significant were sampled at depths ranging between 50 and 500 m, where temperatures decreased only by 1–2 °C. Nonetheless, these species showed a tendency toward an increased zooid size as well. The change in temperature here may not have been large enough to register a significant zooid size change. The fact that zooid size changes were significant for species sampled at a wider range of depths, where temperature gradients were steeper, supports the hypothesis that temperature may have a role in controlling zooid size and is in agreement with previous investigations (e.g., O’Dea and Okamura 1999; Lombardi et al. 2006).

**Table 4:** Range of length, area and Zooid-Shape (ZS) index calculated for each species presented as a percent change from small to large

	Length	Area	ZS index	CV length	CV area
*Bicellarina alderi*	26.9	38.9	12.3	9.1	17.7
*Cabrea ellisi*	12.3	1.8	23.9	10.0	11.8
*Chartella barleei*	18.9	12.5	20.9	10.0	12.5
*Dendrobaenia decorata*	15	19.4	12.8	9.1	14.3
*Dendrobeania fruticosa*	6.9	11.2	17.0	13.4	15.0
*Escharella abysicola*	7.2	10.0	7.6	9.1	5.9
*Escharina boreale*	2.9	6.1	4.2	12.5	22.0
*Porella struma*	11.5	10.0	11.2	10.0	5.9
*Ramphonothus minax*	8.5	16.7	2.1	11.2	6.3
*Sarsiflustra abysicola*	19.2	24.7	1.7	14.3	10.0
*Tricellaria ternata*	1.7	9.1	6.1	12.3	12.5

O’Dea et al. (2007) estimated that a drop in water temperature of 1 °C resulted in a 5% increase in zooid size in *Cupuladria exafragilis*, but even larger differences in zooid size between colonies were recorded when difference in water temperature was at least five degrees (in this case 24 and 29 °C). Similarly, increasing zooid size was recorded in colonies of *Electra pilosa* and *Conopeum reticulum* which grew at different water temperatures (6, 12, 18 and 22 °C; Menons 1972), or in colonies of *Cellepora hyalina* where zooid size was compared between colonies that grew at 8 and 18 °C (Hunter and Hughes 1994). These studies suggest that a water temperature difference of 2 °C across a 50–500 m depth gradient might not be substantial enough to influence differences in zooid characteristics.

Although temperature appear to be an important factor controlling zooid size, there might be other explanations for the observed patterns, such as individual and/or species variability (i.e., some organisms may react differently to environmental conditions). Indeed, the species here investigated belonged to a variety of taxonomic families and reflected a diverse morphology, including encrusting and erect flexible forms. Such differences may have led to different survival strategies and variable metabolic rates. It is also important to note that, in our study, a positive correlation between zooid size and water depth was recorded only for species with erect and flexible colonies. There is some evidence in other groups of animals (e.g., fishes and marine gastropods) that morphology and clad-specific adaptations may determine depth-associated body size changes (Roy 2002; Smith and Brown 2002). To our knowledge, no previous studies on bryozoans investigated such large number of sympatric species with varying morphology and phylogenetic affiliation. Based on our findings, taxon-specific responses to environmental variable or different levels of biological control of zooid size and/or shape are probable.

The existence of some other mechanism of biological control in the regulation of zooid body size became more plausible with the re-analysis of data for the three species where zooid size correlated significantly with depth at a depth-range of 100–1000 m. When restricting the analysis to measurements from specimen occurring at a narrower depth range (100–500 m), there was still a statistically significant correlation between zooid size and water depth, suggesting that responses to environmental factors are species-specific. However, we cannot rule out the fact that temperature differences across the depth-gradient on the Icelandic and Irminger Basins may be insufficient to trigger shifts in zooid size.

We also acknowledge that a wide range of changes in factors such as temperature, light, and food availability would be expressed by difference in within-colony zooid sizes as different zooids bud at different times and are, therefore, exposed to different environmental conditions, including seasonal patterns. O’Dea and Okamura (2000a) suggested that seasonal variations should be reflected in the amount of variability found in within-colony zooid body size. Therefore, we expected to observe larger within-colony CVs in colonies developing in shallower habitats, which were exposed to seasonality. Temperatures differed by approximately 2–3 °C between the spring/summer and autumn/winter seasons at a depth of 100 m, and differences decreased at shallower depths, being undetectable in waters <500 m (Holliday et al. 2006). Yet, CVs calculated for zooid length and surface area for each of the species studied did not change significantly with water depth and trends were quite variable showing both decreases and increases in zooid body size. O’Dea and Okamura (2000a) noticed a small variation in bryozoan zooid size off the Otago Shelf at a depth of 80 m, and from coastal waters off Antarctica, and suggested that low-level seasonal variations in temperature (e.g., 1.5 °C in coastal waters off Antarctica) generated a low-level variation in zooid size.

Our results, combined with data from previous studies, suggest that our study area may experience relatively stable environmental conditions and a level of seasonal variation that is too low to generate significant changes in zooid size with water depth.

Our results have shown that zooid body shape was positively correlated with water depth for just two bryozoan species (*B. alderi*, *C. ellisii*). In addition, only in *B. alderi* all three measurements (zooid length, surface area and body shape) were positively correlated with water depth. To some degree, this is an indication that, zooid body shape is influenced by the same parameters as body length or surface area. In the case of other species, different factors might be responsible for each zooid characteristic. Teasing apart which environmental factor is responsible for which characteristic is difficult without additional investigations. As zooid size could potentially influence colony functioning, leading to differences in feeding rates or waste disposal efficiency, further studies to gain an insight into the factors responsible for zooid size characteristic are recommended.

In the majority of our sampled bryozoan species, zooid length, surface area, body shape and within-colony variations were not correlated with water depth. Based on our general knowledge of environmental factors, it is known that temperature in our study area decreased with depth (Hansen and Østerhus 2000). However, both water temperature and other environmental parameters could vary locally and have no linear correlation with water depth leading, indeed, to the observation of no linear patterns in zooid length, surface area or shape. The effects of water temperature, which we explored at the macro-scale level, could be masked by micro-scale environmental factors. Hageman and Christopher (2014) estimated that about 60% of morphological variance in *Electra pilosa* zooids was due to within-colonies, micro-environmental factors (e.g., position within the colony, and life history of the individual module), while macro-environmental factors (wave energy and nutrient supply) explained only 7.5% of the total variability. Although our study lacks consistent patterns among the currently investigated bryozoan species, we did observe some variability in zooid size characteristics within the study area. Discriminating whether this plasticity is due to genotypic variability, represents the ecophenotypic response of a single species to a given factor (e.g., water temperature), or is due to a combination of factors is very difficult if not impossible to determine based on the available data. More detailed studies combining both field and laboratory approaches are needed to reveal mechanisms controlling bryozoan zooid size. We also suggest that investigations considering the phylogenetic position and taxonomy of the species under investigation may be important.

## Electronic supplementary material

Below is the link to the electronic supplementary material.

Supplementary material 1 (PDF 386 kb)
