# Supplementary material for: Bryozoan zooid size variation across a bathymetric gradient: a case study from the Icelandic shelf and continental slope
Source: Mar Biol. 2017 Sep 7;164(10):197. doi: 10.1007/s00227-017-3231-9 (PMC5589789; doi:10.1007/s00227-017-3231-9)
Supplement: Supplementary file 1 — Supplementary material 1 (PDF 386 kb) [file 227_2017_3231_MOESM1_ESM.pdf]

title: Bryozoan zooid size and shape variability across bathymetric gradient
authors: Anna Stępień1, Piotr Kukliński1, Maria Włodarska-Kowalczuk1, Małgorzata Krzemińska1, Gudmundur Gudmundsson2
affilitions: 1 Institute of Oceanology Polish Academy of Science, Powstańców Warszawy 55, 81-712 Sopot, Poland;
2 The Icelandic Institute of Natural History, Urriðaholtsstræti 6-8, 210 Garðabær, Iceland
corresponding author: astepien@iopan.gda.pl, (48) 635-44-42

Bicellarina alderi																									
sample		3047	3048	3052	3054	3056	3039	2247	2264	2265	2265	2873	2254	2255	2849	2849	2903								
Depth (m)	start of tow	134	130	114	140	100	121	550	544	546	546	554	999	1025	976	976	1066								
	end of tow	141	135	121	141	100	122	552	552	569	569	555	1030	1030	1000	1000	1057								
length (mm)	mean	1.09	1.05	1.04	1.01	1.20	0.94	1.40	1.23	1.26	1.24	1.33	1.57	1.51	1.32	1.40	1.44								
	SD	0.09	0.10	0.10	0.10	0.15	0.17	0.14	0.11	0.13	0.14	0.13	0.15	0.11	0.14	0.13	0.18								
width (mm)	mean	0.35	0.30	0.32	0.30	0.30	0.29	0.35	0.33	0.34	0.33	0.32	0.37	0.39	0.34	0.38	0.38								
	SD	0.02	0.040	0.03	0.03	0.02	0.04	0.026	0.03	0.02	0.04	0.03	0.04	0.04	0.04	0.02	0.04								
Caberea ellisii																									
sample		3039	3047	3052	3054	3056	2946	2947	2948	2710	3061	2355	2358	2398	2512	2512	2873	2873	2892	2893	2893				
Depth (m)	start of tow	121	141	114	140	100	228	227	226	220	221	317	318	346	333	333	554	554	507	513	513				
	end of tow	122	134	121	141	100	229	227	228	230	224	315	330	348	335	335	555	555	575	578	578				
length (mm)	mean	0.36	0.40	0.47	0.42	0.45	0.44	0.46	0.43	0.40	0.29	0.41	0.47	0.40	0.30	0.39	0.53	0.50	0.41	0.54	0.49				
	SD	0.04	0.03	0.05	0.02	0.03	0.03	0.03	0.03	0.04	0.03	0.04	0.05	0.03	0.03	0.02	0.06	0.03	0.04	0.05	0.06				
width (mm)	mean	0.23	0.25	0.27	0.28	0.29	0.25	0.25	0.25	0.24	0.16	0.21	0.25	0.26	0.20	0.18	0.23	0.22	0.19	0.23	0.24				
	SD	0.02	0.03	0.03	0.02	0.03	0.02	0.03	0.02	0.03	0.01	0.02	0.03	0.03	0.02	0.02	0.02	0.02	0.02	0.01	0.02				
Chartella barleei																									
sample		3047	3052	3054	3056	3046	2240	2241	2273	2382	2382	2512	2247	2264	2265	2873	2873	2255	2844	2849	3590				
Depth (m)	start of tow	141	114	140	100	135	307	305	313	302	302	333	550	544	546	554	554	1025	1085	976	1111				
	end of tow	134	121	141	100	140	308	305	316	298	298	335	552	552	569	555	555	1030	1070	1000	1111				
length (mm)	mean	0.81	0.77	0.87	0.82	0.78	0.92	0.89	0.89	0.62	0.84	0.60	0.82	0.84	0.97	0.93	0.93	0.99	0.91	0.79	0.93				
	SD	0.08	0.06	0.07	0.06	0.07	0.14	0.07	0.06	0.05	0.09	0.05	0.08	0.06	0.07	0.10	0.07	0.12	0.07	0.09	0.07				
width	mean	0.29	0.35	0.37	0.34	0.30	0.44	0.39	0.40	0.32	0.36	0.32	0.40	0.39	0.46	0.38	0.35	0.45	0.41	0.45	0.49				
	SD	0.02	0.02	0.03	0.03	0.03	0.06	0.05	0.05	0.03	0.01	0.05	0.05	0.04	0.05	0.04	0.03	0.07	0.05	0.06	0.06				
Dendrobeania decorata																									
sample		3033	3039	3047	3048	3056	3046	2215 k1	2215	2277	2881	2947	2710	2272	2273	2355	2355	2356	2375	2265	2332	2345	2361	2363	2873
Depth (m)	start of tow	139	121	141	130	100	135	213	213	204	219	227	220	316	313	317	317	327	305	546	550	497	498	493	554
	end of tow	141	122	134	135	100	140	219	219	222	220	227	230	319	316	315	315	324	304	569	564	495	494	500	555
length (mm)	mean	1.07	0.99	1.10	0.92	1.04	1.06	1.08	1.16	1.18	0.99	0.94	1.12	1.11	1.04	1.02	1.11	1.08	0.99	1.10	1.22	1.16	1.40	1.39	1.03
	SD	0.12	0.11	0.13	0.08	0.10	0.17	0.13	0.17	0.20	0.09	0.10	0.16	0.15	0.16	0.13	0.10	0.12	0.10	0.10	0.10	0.13	0.16	0.20	0.07
width (mm)	mean	0.23	0.23	0.25	0.30	0.24	0.24	0.21	0.21	0.21	0.21	0.31	0.26	0.22	0.22	0.23	0.24	0.23	0.23	0.22	0.25	0.25	0.28	0.28	0.23
	SD	0.02	0.02	0.01	0.01	0.02	0.03	0.02	0.02	0.02	0.02	0.01	0.02	0.02	0.02	0.02	0.01	0.02	0.02	0.02	0.02	0.02	0.02	0.02	0.01
Dendrobeania fruticosa																									
sample		3268	2207	2204	2533	3046	3046	3047	3039	2948	2947	2946	2868	2710											
Depth (m)	start of tow	63	81	31	77	135	135	141	121	226	227	228	212	220											
	end of tow	64	83	33	76	140	140	134	122	228	227	229	216	230											
length (mm)	mean	0.95	0.78	0.77	0.76	0.73	0.71	0.90	0.85	0.84	0.94	0.86	0.90	0.89											
	SD	0.15	0.06	0.09	0.11	0.09	0.09	0.08	0.08	0.11	0.15	0.12	0.15	0.11											
width (mm)	mean	0.95	0.788	0.77	0.76	0.73	0.71	0.90	0.85	0.84	0.94	0.86	0.90	0.89											
	SD	0.15	0.06	0.09	0.11	0.09	0.09	0.08	0.08	0.11	0.15	0.12	0.15	0.11											
Escharella abyssicola																									
sample		3052	3052	3052	3054	3054	3054	3054	3054	3054	2710	2710	2710	2710	2382	2382	2382	3043	3043	3043	3043	3043			
Depth (m)	start of tow	114	114	114	140	140	140	140	140	140	220	220	220	220	302	302	302	356	356	356	356	356			
	end of tow	121	121	121	141	141	141	141	141	141	230	230	230	230	298	298	298	349	349	349	349	349			
length (mm)	mean	0.79	0.96	1.07	0.89	0.90	0.99	0.91	0.85	0.77	0.86	0.82	0.95	0.91	0.84	0.89	1.03	1.05	0.93	1.09	0.98	1.04			
	SD	0.10	0.09	0.11	0.09	0.11	0.11	0.08	0.08	0.07	0.08	0.07	0.11	0.17	0.11	0.09	0.10	0.11	0.10	0.13	0.12	0.10			
width (mm)	mean	0.53	0.62	0.63	0.59	0.58	0.65	0.64	0.55	0.51	0.58	0.56	0.61	0.56	0.45	0.65	0.67	0.67	0.59	0.65	0.56	0.62			
	SD	0.06	0.09	0.05	0.05	0.05	0.05	0.09	0.06	0.03	0.07	0.05	0.06	0.06	0.05	0.07	0.08	0.07	0.06	0.04	0.06	0.06			
Escharina boreale																									
sample		3047	3054	3054	3054	3275	3275	2719	2719	2719	2872	2724	2724	2724	2724										
Depth (m)	start of tow	141	140	140	140	305	305	300	300	300	549	493	493	493	493										
	end of tow	134	141	141	141	295	295	305	305	305	555	494	494	494	494										
length (mm)	mean	0.66	0.70	0.50	0.63	0.66	0.62	0.69	0.73	0.73	0.76	0.62	0.66	0.69	0.62										
	SD	0.05	0.05	0.06	0.03	0.04	0.04	0.06	0.10	0.06	0.06	0.05	0.06	0.08	0.05										
width (mm)	mean	0.42	0.45	0.65	0.52	0.48	0.43	0.49	0.46	0.53	0.57	0.43	0.51	0.46	0.48										
	SD	0.05	0.05	0.06	0.04	0.03	0.03	0.052	0.05	0.08	0.09	0.07	0.09	0.07	0.07										
Porella struma																									
sample		2881	2884	2946	2710	2719	2398	2889	3552	3552	3554	3555	2247	2873	2892	2893	2893								
Depth (m)	start of tow	219	229	228	220	300	346	332	300	300	304	300	550	554	507	513	513								
	end of tow	220	232	229	230	305	348	332	304	304	308	307	552	555	575	578	578								
length (mm)	mean	0.74	0.85	0.75	0.74	0.77	0.86	0.83	0.96	0.90	0.87	0.83	1.02	0.95	0.71	0.89	0.94								
	SD	0.09	0.07	0.08	0.07	0.07	0.09	0.07	0.10	0.08	0.08	0.05	0.10	0.09	0.04	0.07	0.11								
width (mm)	mean	0.42	0.53	0.42	0.48	0.51	0.49	0.50	0.45	0.54	0.53	0.50	0.52	0.49	0.42	0.47	0.44								
	SD	0.05	0.04	0.06	0.06	0.05	0.06	0.06	0.06	0.07	0.06	0.07	0.09	0.05	0.05	0.04	0.05								
Ramphonotus minax																									
sample		3048	3046	3046	3046	2277	2946	2947	2948	2710	2356	3275	3557	3558	3559	2719	2265	2265	2872	2873	3014	2849	3002	3002	3002
Depth (m)	start of tow	130	135	135	135	204	228	227	226	220	327	305	334	336	337	300	546	546	549	554	504	976	998	998	998
	end of tow	135	140	140	140	222	229	227	228	230	324	295	337	337	338	305	569	569	555	555	505	1000	968	968	968
length	mean	0.57	0.57	0.62	0.59	0.57	0.54	0.55	0.50	0.54	0.60	0.61	0.52	0.57	0.63	0.57	0.55	0.58	0.53	0.49	0.47	0.58	0.50	0.55	0.56
	SD	0.03	0.04	0.03	0.05	0.06	0.05	0.04	0.03	0.05	0.05	0.04	0.05	0.06	0.06	0.09	0.04	0.05	0.04	0.06	0.03	0.06	0.04	0.04	0.04
width	mean	0.37	0.40	0.48	0.37	0.42	0.44	0.45	0.36	0.45	0.43	0.40	0.42	0.39	0.46	0.35	0.41	0.45	0.40	0.40	0.34	0.45	0.33	0.33	0.39
	SD	0.02	0.03	0.05	0.04	0.05	0.05	0.03	0.03	0.03	0.04	0.04	0.04	0.04	0.03	0.03	0.03	0.04	0.04	0.04	0.02	0.04	0.02	0.04	0.04
Sarsiflustra abyssicola																									
sample		2889	3552	3552	3554	3558	2247	2265	2873	2892	2893	2254	2844	2844	2849	2903	2904								
Depth (m)	start of tow	332	300	300	304	336	550	546	554	507	513	999	1085	1085	976	1066	1057								
	end of tow	332	304	304		337	-	-	555	575	578	1030	1070	1070	1000	-	-								
length (mm)	mean	1.24	1.11	1.10	1.11	1.08	1.19	1.23	1.10	1.23	1.22	1.19	1.32	1.06	1.04	1.21	1.26								
	SD	0.17	0.07	0.18	0.20	0.19	0.16	0.07	0.14	0.17	0.18	0.16	0.16	0.09	0.11	0.18	0.16								
width (mm)	mean	0.47	0.48	0.44	0.41	0.44	0.49	0.64	0.44	0.51	0.47	0.48	0.53	0.48	0.43	0.50	0.55								
	SD	0.05	0.03	0.07	0.06	0.04	0.05	0.04	0.05	0.05	0.05	0.02	0.04	0.04	0.039	0.05	0.04								
Tricellaria ternata																									
sample		2884	2884	2946	2947	3561	3552	3554	3554	3558	3558	2873	2873	2892	2893										
Depth (m)	start of tow	229	229	228	227	230	300	308	308	336	336	554	554	507	513										
	end of tow	232	232	229	227	236	304			337	337	555	555	575	578										
length (mm)	mean	0.61	0.58	0.70	0.60	0.57	0.57	0.64	0.58	0.56	0.54	0.63	0.60	0.58	0.52										
	SD	0.06	0.05	0.05	0.04	0.03	0.02	0.10	0.06	0.06	0.03	0.07	0.06	0.06	0.02										
width (mm)	mean	0.18	0.17	0.21	0.20	0.18	0.16	0.19	0.16	0.17	0.17	0.17	0.18	0.16	0.17										
	SD	0.01	0.01	0.02	0.02	0.01	0.01	0.03	0.01	0.02	0.02	0.01	0.02	0.02	0.01										
